# Cloning and Characterization of GDSL Esterases from *Bacillus paralicheniformis* T7

**DOI:** 10.3390/biology15030276

**Published:** 2026-02-03

**Authors:** Arman Mussakhmetov, Magzhan Astrakhanov, Dmitriy Silayev, Bekbolat Khassenov

**Affiliations:** 1 “National Center for Biotechnology” Ltd., Astana 010000, Kazakhstan; mussakhmetov@biocenter.kz (A.M.);; 2 Faculty of Natural Science, L.N. Gumilyev Eurasian National University, Astana 010000, Kazakhstan; 3 “GenLab” Ltd., Astana 010000, Kazakhstan

**Keywords:** esterases, *Bacillus*, enzyme, cloning, strain, recombinant

## Abstract

Esterases catalyze the hydrolysis and transesterification of short-chain fatty acid esters, and microbial esterases are used in the production of biofuels, cosmetics, food, and pharmaceuticals. The soil strain *Bacillus paralicheniformis* T7 secretes enzymes with esterase activity; however, many bacterial enzymes remain insufficiently studied. Therefore, this study aimed to identify and characterize novel GDSL esterases produced by *B. paralicheniformis*. Protein mass spectrometry, combined with proteomics and genomics, identified genes encoding two GDSL esterases, which were cloned into the pET-28c(+) vector. The resulting proteins were obtained in *Escherichia coli* BL21(DE3) as the recombinant esterases rEST-24 and rEST-28. These recombinant GDSL esterases showed maximum activity at 40 °C and pH 7.0. Moreover, Ca^2+^, Zn^2+^, Cu^2+^, and Fe^2+^ ions inhibited their activity, and rEST-28 was resistant to the detergents Tween-20, Tween-80, and Triton X-100. High-yield esterase activity was detected in bacteria cultured on feather medium and nutrient broth, and submerged fermentation of the *B. paralicheniformis* T7 strain on feather medium enabled the production of an esterase extract exhibiting activity of 17,618 ± 610 U/g. These results suggest that the *B. paralicheniformis* T7 strain can produce esterases and shows promising potential for application in technologies that degrade fatty acid esters using hydrolytic enzymes.

## Simple Summary

Various contemporary biotechnological and industrial procedures rely on enzymes that effectively degrade lipids and associated substances. However, enzymes that maintain activity across diverse conditions remain scarce. Microorganisms are a key source of these enzymes; however, numerous bacterial enzymes remain insufficiently studied. In this study, we focused on the identification and characterization of novel enzymes derived from bacteria isolated from natural environments. We specifically examined a soil bacterium, *Bacillus paralicheniformis* T7, which synthesizes enzymes known as esterases. Two esterases produced by this bacterium were identified, isolated, and comprehensively examined. The study findings indicated that *B. paralicheniformis* T7 represents a promising biological source of enzymes for sustainable technologies that depend on environmentally benign degradation of fat-based materials.

## 1. Introduction

Esterases are ubiquitous enzymes found in all living organisms, including animals, plants, and microorganisms [1]. These enzymes participate in various biological processes, including cell signalling, remodelling, phosphorus metabolism, detoxification of natural and synthetic substances, and the synthesis and breakdown of biomolecules and polymers, such as nucleic acids, lipids, and esters [2]. This class of hydrolase enzymes, particularly EC 3.1.1.1, catalyse the hydrolysis and transesterification of fatty acid esters [3].

In terms of structural organisation, these enzymes are serine hydrolase group members and have a characteristic α/β-hydrolase fold [4]. Their catalytic mechanism is based on an amino acid triad comprising serine, histidine, and aspartate or glutamate, which is located in the active centre [3]. For example, the amino acid residues Ser77, His76, and Gly103 of *Bacillus subtilis* E9 esterase interact with the ligand, thus providing the enzyme with the necessary activity [5]. Unlike lipases, esterases exhibit differences in kinetic properties and substrate specificity [6]. Specifically, esterases predominantly hydrolyse water-soluble esters of fatty acids with short hydrocarbon chains (<10 carbon atoms), whereas lipases act on insoluble triglycerides with long-chain residues (≥10 carbon atoms) [7].

In terms of practical applications, microbial esterases have high value owing to their high activity, stability over a wide range of temperatures and pH values, and resistance to solvents and detergents. Hence, microbial esterases are used in industries such as biofuel production, food processing, pesticide removal, pharmaceutical synthesis and cosmetics [8,9].

Among the estarase-producing microorganisms of industrial importance are bacteria of the genera *Aeromonas*, *Streptomyces*, *Pseudomonas*, and *Bacillus* [10,11,12]. In particular, esterases of bacillary origin have attracted attention from researchers. The specificity and enantioselectivity of *Bacillus* esterases and lipases render them valuable tools for synthesizing optically pure compounds, such as D-methyl lactates, which are used to produce many pharmaceuticals and chiral chemicals [13,14]. Furthermore, these enzymes are used to prepare compounds with high enantiomeric excess in a stereoselective manner, thereby reducing the costs of subsequent purification and improving the overall efficiency of pharmaceutical manufacturing processes [14]. Moreover, the high substrate specificity and robust catalytic profiles of *Bacillus* lipases facilitate their use in drug metabolism studies to mimic human metabolic pathways and evaluate potential drug interactions [15].

Notably, GDSL esterases, which are a subfamily of serine hydrolases, are distinguished among esterases by a characteristic motif (GDSxxDxG) near the N-terminus, belonging to the SGNH superfamily. Unlike carboxyl esterases, GDSL esterases often have multiple catalytic blocks, which determine a wide range of substrate specificity and regio- and enantioselectivity [16]. Thus, such enzymes are used as biocatalysts in the synthesis of esters and aromatic compounds, in the biodegradation of lipophilic pollutants, and in the food and oil processing industries [10,17,18]. Suitably, representatives of the genus *Bacillus* possess functional GDSL esterases with desirable industrial properties, such as thermal and alkaline stability, and the ability to function in the presence of detergents [17]. Specifically, esterase activity was previously detected in the secretory extract of *Bacillus paralicheniformis* T7 isolated from soil [19,20]. Although, bacteria are a key source of esterases, many bacterial enzymes remain insufficiently studied.

Therefore, this study aimed to identify and characterize novel GDSL esterases produced by the *Bacillus paralicheniformis* T7 strain. The esterase genes were cloned and expressed in *Escherichia coli* cells. Recombinant enzymes were then isolated and purified, and their biochemical properties, substrate specificity, and kinetic parameters were determined. Moreover, various culture media were tested to increase the esterase yield when culturing the *B. paralicheniformis* T7 strain. The strain was then fermented in a bioreactor to produce a dried preparation with esterase activity.

## 2. Materials and Methods

### 2.1. Reagents, Media, and Enzymes

All chemical reagents were obtained from Sigma-Aldrich (St. Louis, MO, USA) and AppliChem (Darmstadt, Germany). The following media were used to culture the *B. paralicheniformis* and *E. coli* strains: lysogeny broth (pH 7.0) comprising 0.5% yeast extract (Condalab, Madrid, Spain), 1% tryptone (Sigma-Aldrich), and 0.5% NaCl (Sigma-Aldrich); nutrient broth (HiMedia, Mumbai, India); feather medium with yeast extract (pH 7.0) comprising 0.03% NaH_2_PO_4_, 0.035% Na_2_HPO_4_, 0.2% yeast extract, and 0.75% feather powder; salt medium with Tween-20 (pH 7.0) comprising 0.01% (NH_4_)_2_SO_4_, 0.016% K_2_HPO_4_, 0.02% KH_2_PO_4_, 0.002% CaCl_2_, 0.02% MgSO_4_, 0.005% ZnSO_4_, 0.005% MnSO_4_, 0.001% FeSO_4_, and 1% Tween 20; and yeast extract peptone (YEPT; pH 7.0) comprising 0.3% yeast extract, 0.5% peptone (TM Media, Delhi, India), and 1% tributyrin.

### 2.2. Strains, Vectors, and Oligonucleotides

The following bacterial strains were used: *Bacillus paralicheniformis* T7, which was isolated from soil [21], and *E. coli* strains DH5α (Promega, Madison, WI, USA) and BL21(DE3) (Novagen, Madison, WI, USA). The pET-28c(+) vector (Novagen) was used to clone the esterase genes and produce recombinant proteins in *E. coli* cells. The oligonucleotides used to clone the esterase genes and sequence the insert are listed in Table 1.

### 2.3. Cloning of Esterase Genes into Vectors

The *B. paralicheniformis* T7 was cultured in 5 mL of nutrient broth in a shaking incubator at 37 °C and 150 rpm for 18 h. Thereafter, the cells were collected via centrifugation at 6000× *g* and 4 °C for 7 min. Subsequently, the genomic DNA was isolated using a Monarch Nucleic Acid Purification Kit (New England Biolabs, Ipswich, MA, USA). The *est-24* and *est-28* esterase genes were then amplified from the genomic DNA via PCR using the oligonucleotides EST-24fw/EST-24rv and EST-28fw/EST-28rv. The amplified fragments were cloned into the pET-28c(+) vector at the NdeI and BamHI sites using appropriate restriction endonucleases in 2X Tango buffer (Thermo Fisher Scientific, Vilnius, Lithuania). The T7 region of the resulting construct was sequenced using the Sanger method [22] and BigDyeTerminator v3.1 (Thermo Fisher Scientific). The sequenced DNA fragments were then separated using an ABI 3730 xl automated sequencer (Applied Biosystems, Foster City, CA, USA). The resulting pET-28/EST-24 and pET-28/EST-28 plasmid vectors were produced in *E. coli* DH5α cells and isolated using a GeneJET Plasmid Miniprep Kit (Thermo Fisher Scientific).

### 2.4. Esterase Expression and Purification of Recombinant Proteins

Competent *E. coli* BL21(DE3) cells were transformed with the pET-28/EST-24 and pET-28/EST-28 vectors. Transformation was performed using a MicroPulser electroporator (Bio-Rad Laboratories, Hercules, CA, USA). The transformant clones were then selected on Luria–Bertani (LB) agar containing kanamycin (50 μg/mL) and cultured in LB broth containing kanamycin until an optical density of 0.6 at a wavelength of 600 nm was achieved. Thereafter, isopropyl β-d-1-thiogalactopyranoside (0.5 mM) was added, and protein induction was carried out for 16 h at 18 °C.

The cells were collected via centrifugation at 6000× *g* for 6 min and lysed using a Ruptor 4000 (OMNI, Kennesaw, GA, USA) sonicator. The water-soluble fraction was then separated via centrifugation at 40,000× *g* for 1 h at 4 °C. Thereafter, the fraction was applied to a column with nickel-nitrilotriacetic acid (Ni-NTA) agarose beads (Invitrogen, Carlsbad, CA, USA), which had been pre-equilibrated with a buffer containing 20 mM Tris-HCl (pH 8.0), 500 mM NaCl, and 20 mM imidazole. Subsequently, the recombinant protein was eluted using a stepwise increase in imidazole concentration: 100, 150, 200, and 250 mM in a buffer containing 20 mM Tris-HCl (pH 8.0) and 500 mM NaCl. Fractions were analysed via sodium dodecyl sulfate (SDS)-polyacrylamide gel electrophoresis (PAGE). The fractions containing target proteins were concentrated and buffered using a protein concentrator (Pierce™ Protein Concentrators PES, Thermo Fisher Scientific) with a cutoff of 10,000. The protein concentrations were measured and used in subsequent experiments.

### 2.5. SDS-PAGE

Electrophoretic separation of proteins via SDS-PAGE was performed in a 12% polyacrylamide gel according to the Laemmli method [23] in a MiniProtean IV cell (Bio-Rad Laboratories). A Pierce™ Unstained Protein MW Marker (Thermo Fisher Scientific, cat. #26610) was used as a molecular weight marker for the proteins.

### 2.6. Protein Concentration Measurements

The protein concentration was determined using the Bradford method [24] with Protein Assay Dye Reagent (Bio-Rad Laboratories), and bovine serum albumin was used as the standard. Briefly, 100 μL of Bradford reagent was mixed with 860 μL of 10% phospahte buffered saline (PBS) solution containing 1% glycerol, and 40 μL of the sample was added. The solution was shaken and incubated for 2 min at room temperature. The optical density was then measured using a spectrophotometer at a wavelength of 595 nm.

### 2.7. Enzyme Activity Assays

Esterase activity was measured according to Zhao et al. [25]. Briefly, 100 μL of the sample and 50 μL of the substrate (10 mM p-nitrophenyl acetate dissolved in isopropanol) were added to 350 μL of 50 mM sodium phosphate buffer at pH 7.0. The mixture was then incubated at 40 °C for 10 min, and the reaction was stopped by adding 1 mL of ethanol. The solution was then centrifuged at 1000× *g* for 3 min, and the absorbance was measured at 410 nm using a UV1900i spectrophotometer (Shimadzu, Kyoto, Japan). One unit of esterase activity corresponded to the formation of 1 μmol of 4-nitrophenol (*p*NP) per minute under these conditions.

### 2.8. Effect of Temperature and pH on Enzyme Activity and Stability of Recombinant Esterases

Enzyme activity was measured within a pH range of 3.0–11.0. The following buffer systems were used: citrate buffer (pH 3.0–5.0), sodium phosphate buffer (pH 6.0–7.0), and glycine-NaOH buffer (pH 8.0–11.0). The obtained values were converted to relative units, with the maximum value set at 100%. For the pH stability experiments, the enzyme extract was pre-incubated for 2 h at 23 °C in 10 mM citrate buffer at pH 4.0 and 6.0 and in 50 mM Tris-HCl buffer at pH 8.0 and 10.0. The residual activity was then measured in 50 mM sodium phosphate buffer at pH 7.0. The results were expressed as a percentage of the activity of the unpreincubated extract, set at 100%. The effect of temperature on esterase activity was assessed in the range of 30–80 °C in intervals of 5 °C. The maximum enzymatic activity was taken as 100%. To analyse the thermal stability, the enzyme extract was pre-incubated for 5 h at 40, 50, 60 or 70 °C in 50 mM Tris-HCl buffer solution (pH 7.0). The residual activity was then measured at 40 °C in 50 mM sodium phosphate-buffered solution (pH 7.0). The results were expressed as a percentage of the activity of the extract not subjected to thermal incubation. All experiments were performed in triplicate.

### 2.9. Effect of Metal Ions and Detergents on Esterase Activity

The effect of metal ions on esterase activity was determined using the following ions: Ca^2+^, Mg^2+^, Mn^2+^, Cu^2+^, Zn^2+^, and Fe^2+^. The effect of the metal ions, the reducing agents β-mercaptoethanol and dithiothreitol (DTT), the inhibitor phenylmethylsulfonyl fluoride, the chelating agent ethylenediaminetetraacetic acid (EDTA), and the detergents Tween-20, Tween-80, Triton X-100, and SDS on enzymatic activity was determined by adding the reagents to the reaction mixture. Enzymatic activity was then assessed at 40 °C in a 50 mM sodium phosphate-buffered solution (pH 7.0). Activity values in the absence of metal ions and chemicals were considered 100% activity.

### 2.10. Substrate Specificity of Recombinant Esterases

The substrate specificity of the recombinant esterases was determined using *p*NP esters with different acyl chain lengths (C2–C18). The reaction was carried out in 50 phosphate buffer (pH 7.0) at 40 °C for 10 min and the concentration of each substrate was 10 mM. The following substrates were used to determine substrate specificity: 4-nitrophenyl acetate, 4-nytrophenyl octanoate (Thermo Fisher Scientific), 4-nitrophenyl palmitate, 4-nitrophenyl dodecanoate (Sigma-Adlrich), 4-nitrophenyl decanoate, 4-nitrophenyl stearate (BLDpharm, Songjiang, Shanghai, China), 4-nitrophenyl butyrate (Cayman chemical company, Ann Arbor, MI, USA), 4-nytrophenyl myristate, and 4-nytrophenyl hexanoate (Apollo Scientific, Stockport, UK).

### 2.11. Kinetic Parameters and Specific Activity of Recombinant Esterases

The kinetic parameters were measured using an enzymatic reaction to cleave p-nitrophenyl acetate at concentrations of 0.01–2.00 mM in a 50 mM sodium phosphate-buffered solution at pH 7.0 and a temperature of 40 °C. The Michaelis constant (K_m_) and maximum velocity (V_max_) values were calculated using the Michaelis–Menten equation [26]. To determine the K_m_ and catalytic rate (K_cat_) constants, the linear velocity was measured, and the constants were determined using the one-site binding (hyperbolic) model with GraphPad Prism 8.0.1 (GraphPad Software, San Diego, CA, USA). The specific activity was measured under optimal conditions with p-nitrophenyl acetate serving as the substrate for rEST-28 and p-nitrophenyl decanoate for rEST-24.

### 2.12. Effect of Culture Media on Esterase Activity

*Bacillus paralicheniformis* T7 cultures were grown independently in four 1 L flasks in a KS4000i Control shaker incubator (IKA, Staufen im Breisgau, Germany). To obtain a total inoculum, the culture was grown in 50 mL of lysogeny broth overnight at 37 °C and 150 rpm. The cells were collected via centrifugation at 6000× *g* at 4 °C for 7 min. The supernatant was removed, and the pellet washed in 1 mL of PBS. This process was repeated, and the supernatant removed again. Thereafter, the pellet was suspended in 1 mL of PBS, and 200 μL of the suspension was inoculated into 200 mL of sterile nutrient broth, feather medium with yeast extract, salt medium with Tween 20, and YEPT medium. The cultures were then grown at 37 °C and 150 rpm for 102 h. After 6, 24, 30, 48, 54, 72, 78, 96, and 102 h, 1 mL samples were taken and centrifuged at 10,000× *g* for 5 min at 4 °C. The esterase activity of the resulting supernatants was then measured using p-nitrophenyl acetate.

### 2.13. Effect of Fermentation on Esterase Activity

The ability of *B. paralicheniformis* T7 to produce esterases during submerged fermentation was tested using a 10 L Biostat bioreactor (Sartorius, Göttingen, Germany). Submerged fermentation was carried out according to Akishev et al. [27]. Briefly, a single colony was inoculated into 5 mL of nutrient broth, cultured at 37 °C in a shaker incubator at 180 rpm for 18 h, and then transferred to 200 mL of feather medium supplemented with 0.2% yeast extract. This was grown under the same conditions for a further 24 h. Thereafter, the culture was inoculated into 6 L of sterile feather medium with 0.2% yeast extract in a 10 L fermenter. The submerged fermentation conditions were as follows: temperature, 37 °C; stirring, 450 rpm; and aeration, 6–10 L/min. Subsequently, the strain culture was freed from the cells and substrate residues via centrifugation at 11,000× *g*, and sterilised via microfiltration using a UPIRO-018 benchtop filter apparatus (Vladisart, Vladimir, Russia) and an MKM46020 polyethersulfone membrane module (Vladisart) with a cutoff threshold of 0.22 µm. The clarified cultures were frozen at −80 °C in a U570 ultra-low freezer (New Brunswick Scientific, Enfield, CT, USA) and then dried under vacuum (0.028 mbar) at −90 °C in a BETA 2-8 LDplus lyophilizator (Christ, Osterode, Germany) for 48 h. The lyophilised enzyme preparation was ground into a powder, and the esterase activity was measured using p-nitrophenyl acetate.

### 2.14. Sequence Analysis

To compare conserved motifs in the amino acid sequence, peptide sequences of bacterial SGNH esterases/lipases containing the GDSL motif were searched in the National Center for Biotechnology Information and Protein Data Bank databases. Multiple sequence alignment was performed using Clone Manager software7, version 7.11 (Sci-Ed Software, Westminnster, CO, USA). The secondary structure prediction and the analysis of multiple sequence alignments were conducted using ESPript 3.0 (https://espript.ibcp.fr/ESPript/ESPript/index.php, accessed on 29 January 2026). Multiple sequence alignment of Est-24 was performed using the sequences of Lipase Lip_vut3 from the Goat Rumen metagenome and GDSL-type esterase/lipase family proteins (pdb_00006nkd) from *Bacillus halotolerans* (Accession No. WP_326139525), *B. haynesii* (Accession No. WP_268350347), *B. swezeyi* (Accession No. WP_414557567), *B. velezensis* (Accession No. WP_413544555), and *B. safensis* (Accession No. WP_075622364), which were identical to Est-24 by 94%, 57%, 95%, 82%, 53%, and 45%, respectively. Multiple sequence alignment of Est-28 was performed using the sequences of GDSL-type esterase/lipase family proteins из multispecies of *Bacillus* (Accession No. WP_035428200), *B. swezeyi* (Accession No. WP_414555771), *B. maginnsis* (Accession No. WP_435302229), multispecies of *Bacillus* (Accession No. WP_010335020), and *B. pumilus* (Accession No. WP_260980731), which were identical to Est-28 84%, 89%, 59%, 65%, and 60%, respectively.

### 2.15. Software Tools, Bioinformatics, and Statistical Analysis

Capillary sequencing chromatograms were analyzed using Vector NTI Advance 11 software (Thermo Fisher Scientific). The online SignalP 5.0 platform was used to analyze the protein sequence for the presence of a secretory peptide (http://www.cbs.dtu.dk/services/SignalP/, accessed on 29 January 2026). All activity experiments were performed in triplicate. Enzyme activity data were obtained from independent assays, and mean values, standard deviations (SD), and *p*-values were calculated using GraphPad Prism version 8.0.1 (GraphPad Software, USA). All data are presented as the mean ± SD (n = 3).

## 3. Results

### 3.1. Cloning of Esterases est-24 and est-28 from Bacillus paralicheniformis T7

Oligonucleotides were designed to amplify the GDSL esterase genes *est-24* (636 bp) and *est-28* (678 bp) based on the complete genome sequence of *B. paralicheniformis* T7 (GenBank accession no. CP124861). These genes were then cloned into the rEST-28c(+) vector. The recombinant proteins contained a 6×His-tag at the N-terminus of the open reading frame comprising 232 and 247 amino acids, respectively. The calculated molecular masses and predicted pI values for the rEST-24 and rEST-28 proteins were 26.1 and 27.9 kDa and 6.05 and 9.56, respectively.

### 3.2. Purification of rEST-24 and rEST-28 Esterases

**Figure 1:**
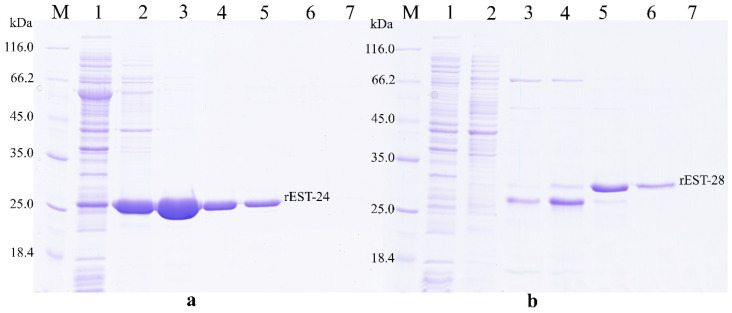
Sodium dodecyl sulfate-polyacrylamide gel electrophoresis (SDS-PAGE) electrophoresis following the chromatographic purification of rEST-24 (**a**) and rEST-28 (**b**) on nickel-nitrilotriacetic acid (Ni-NTA). Line M, protein marker (Thermo Scientific, cat. # 26610); line 1 the supernatant passed through colomn with Ni-NTA, lines 2–7 eluates with 20, 80, 100, 150, 250, and 500, respectively.

The expression of rEST-24 and rEST-28 in *E. coli* BL21(DE3) cells revealed that the recombinant esterases were present in both the water-soluble fraction and the pellet, where they accumulated as inclusion bodies. During chromatographic purification, rEST-24 and rEST-28 were eluted from Ni-NTA at an imidazole concentration of 250 mM (Figure 1). The yields of the purified enzymes were 42 mg and 2.9 mg per litre of induced culture, respectively.

### 3.3. Effects of pH and Temperature on the Enzymatic Activity of rEST-24 and rEST-28

**Figure 2:**
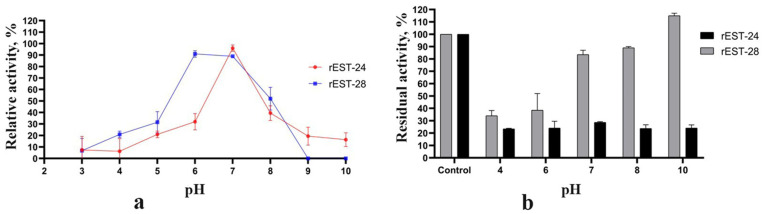
Effect of pH on esterase activity (**a**) and stability (**b**) of rEST-24 and rEST-28. Values are presented as mean ± standard deviation (SD) and are representative data obtained in triplicate assays.

The effects of pH on the activity of rEST-24 and rEST-28 was evaluated using p-nitrophenyl acetate as a substrate in the pH range of 3.0–10.0. The rEST-24 esterase exhibited maximum activity at pH 7.0 only, whereas rEST-28 exhibited maximum activity at pH 6.0–7.0 (Figure 2a). Moreover, assessment of pH stability during enzyme incubation at pH 4.0, 6.0, 7.0, 8.0 and 10.0 showed that the residual activity of rEST-24 did not exceed 30%, whereas rEST-28 retained over 80% of its esterase activity at pH values of >7.0. Furthermore, incubation of rEST-28 at pH 10.0 increased esterase activity by 15% (Figure 2b).

**Figure 3:**
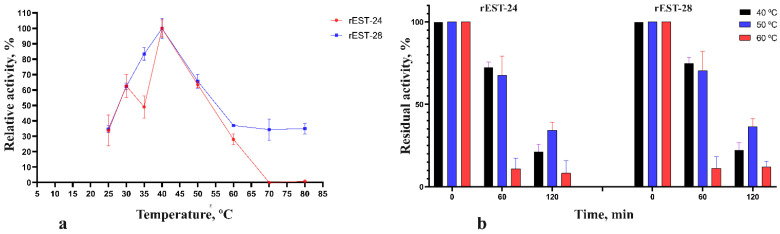
Effect of temperature on esterase activity (**a**) and stability (**b**) of rEST-24 and rEST-28. Values are presented as mean ± standard deviation (SD) and are representative data obtained in triplicate assays.

In addition, the effects of temperature on the activity of rEST-24 and rEST-28 were investigated at temperatures ranging from 25 to 80 °C. Overall, the enzymes exhibited maximum activity at 40 °C (Figure 3a); however, within the temperature range of 30–50 °C, both rEST-24 and rEST-28 demonstrated 50% of their maximum activity. At 60 °C, their relative esterase activities were 37% and 28%, respectively. Notably, rEST-24 was no longer active at 70 °C, whereas rEST-28 exhibited over 30% of its maximum activity at this temperature. Figure 3b illustrates the residual activity of rEST-24 and rEST-28 following a 120 min incubation period at temperatures of 40, 50, and 60 °C. Both enzymes exhibited similar temperature stability. After a 2 h incubation, the residual activity of rEST-24 was 23.5, 31.5, and 12.5% at 40, 50, and 60 °C, respectively, whereas that of rEST-28 was 20, 33.5, and 13.5%, respectively.

### 3.4. Effects of Metal Ions, Detergents, Reducing Agents, and Inhibitors on Stability of rEST-24 and rEST-28

The effects of metal ions on the activity of rEST-24 and rEST-28 were investigated and revealed that a concentration of 10 mM Ca^2+^ (Table 2) reduced the esterase activity of rEST-24 and rEST-28 by 50% and 86%, respectively. Unlike rEST-28, which was sensitive to Mg^2+^ ions, rEST-24 esterase was tolerant of Mg^2+^. Moreover, Mn^2+^ increased the esterase activity of rEST-24 and rEST-28 by 26% and 62%, respectively, whereas Cu^2+^ completely inhibited rEST-24 activity and reduced rEST-28 activity by 68%. Zn^2+^ ions reduced the esterase activity of rEST-24 and rEST-28 by 77% and 67%, respectively, whereas Fe^2+^ ions inhibited rEST-24 activity and reduced rEST-28 activity by 66%. The addition of 10 mM EDTA reduced esterase activity by more than 40%. DTT and β-mercaptoethanol markedly inhibited esterase activity, thereby reducing rEST-28 activity to zero when added. The addition of the non-ionic detergents Tween-20, Tween-80, and Triton X-100 reduced esterase activity by 10–54%. Furthermore, SDS reduced the esterase activity of rEST-24 and rEST-28 by 70% and 11%, respectively.

### 3.5. Substrate Specificity and Kinetic Characteristics

The substrate specificity of EST-24 and EST-28 was studied using p-nitrophenyls with different acyl chains: from C2 (*p*NP-acetate) to C18 (*p*NP-stearate). Table 3 shows the specific activity of these substrates.

The highest activity of rEST-24 was observed for *p*NPD, whereas the maximum activity of rEST-28 was observed for *p*NPA. The K_m_ values for rEST-24 and rEST-28 for *p*NPD and *p*NPA were 11.5 and 26.18 mM, respectively. The V_max_ value was 23.2 and 52.37 U/mg for rEST-24 and rEST-28, respectively. The K_kat_ value was 0.18 s^−1^ and 1.74 s^−1^ for rEST-24 and rEST-28, respectively.

### 3.6. Effect of Culture Media and Fermentation on Esterase Activity

**Figure 4:**
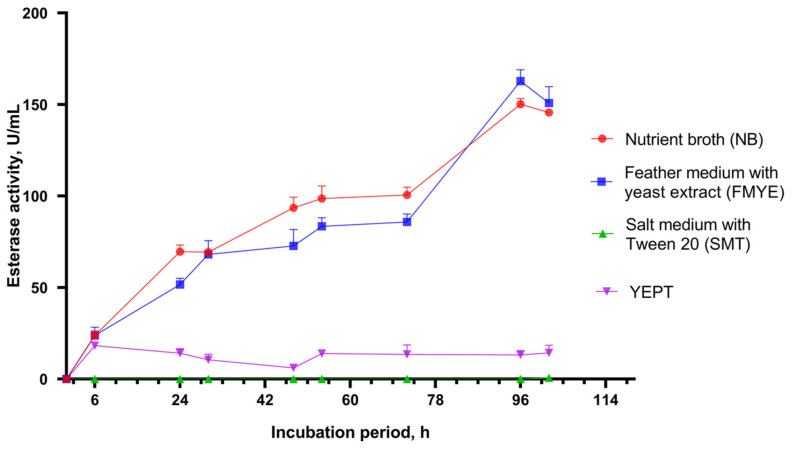
Flask culture of *Bacillus paralicheniformis* T7 in nutrient broth (NB), feather medium with yeast extract (FMYE), salt medium with Tween 20 (SMT), and yeast extract peptone medium (YEPT). Values are presented as the mean ± standard deviation (SD) and are representative data obtained in triplicate assays.

The wild strain of *B. paralicheniformis* T7 was cultured for five days in four different media: nutrient broth, feather medium with yeast extract, salt medium with Tween 20, and YEPT. Figure 4 shows how the esterase activity of the culture depends on the cultivation time. The highest activity was observed in the feather medium, with an esterase activity of 163 ± 6 U/mL after 96 h. Cultivation in nutrient broth also yielded the highest activity after 96 h (150 ± 3 U/mL). No esterase activity was observed in the salt medium with Tween 20 or YEPT (Figure 4).

In addition, the *B. paralicheniformis* T7 strain was cultivated via submerged fermentation in a 10 L bioreactor using feather medium with yeast extract. The culture fluid was then lyophilized at −52 °C under vacuum for 63 h to obtain the enzyme preparation. The esterase activity of the preparation was 17,618 ± 610 U/g.

## 4. Discussion

Microorganisms isolated from the environment are a source of a various enzymes of great industrial importance [28]. Particular attention is given to the genus *Bacillus*, which has several advantages, including high protein synthesis, the ability to secrete enzymes, and potential industrial applications [29]. Esterases are in high demand in various fields of microbial enzyme research, including biodiesel production, advanced food processing, the removal of chemical pesticides from fruit and vegetables, the cosmetics industry, chemical synthesis, and pharmaceutical production [8,9,30]. In particular, GDSL esterases have attracted considerable interest owing to their unique structural features and catalytic properties that distinguish them from classical esterases and lipases. One such distinctive feature is their conserved N-terminal motif, which distinguishes them from classical lipases and renders them members of the SGNH hydrolase superfamily. A detailed analysis of the enzyme sequences revealed the presence of the following four conserved sequence blocks containing invariant residues: serine in block I, glycine in block II, asparagine in block III, and histidine in block V [17]. Collectively, these blocks form a highly conserved catalytic centre, which is crucial for efficient substrate hydrolysis. Unlike classical lipases, which typically contain a Gly-X-Ser-X-Gly motif, *Bacillus* GDSL esterases are characterised by a GDSL tetrapeptide, which is often embedded within a larger conserved pentapeptide, such as GLSLG [31]. The three-dimensional structure of these enzymes is determined by the α/β-hydrolase fold, which is renowned for its structural stability and versatility. Molecular modelling of *Bacillus* GDSL esterases, based on homologous structures in *Bacillus* and related bacteria, reveals a central β-sheet surrounded by α-helices. This arrangement creates a stable scaffold that supports the catalytic triad and facilitates the formation of a narrow active site pocket [17,32].

**Figure 5:**
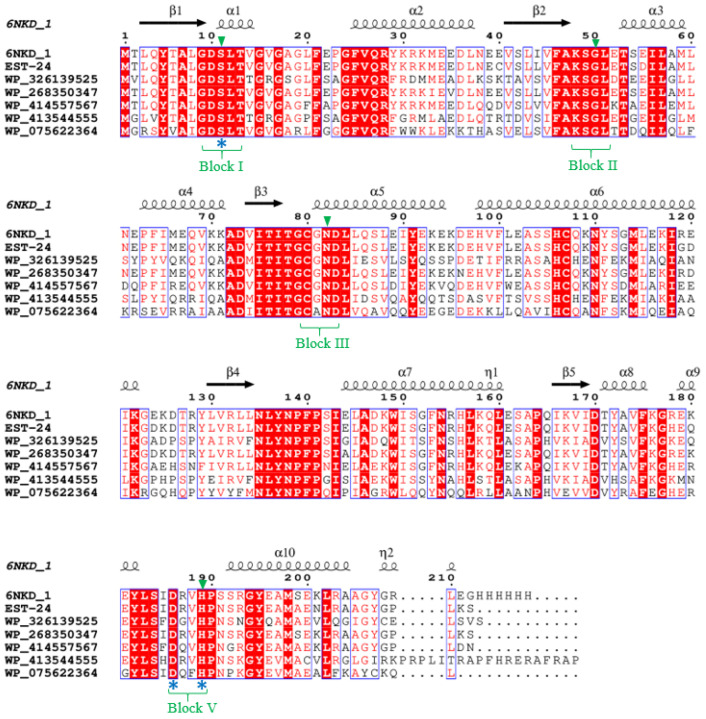
Amino acid sequence alignment analysis of EST-24. Sequences retrieved from National Center for Biotechnology Information (NCBI) server and aligned in Clustal W and rendered using ESPript output. 6MKD_1—Lipase Lip_vut3 from Goat Rumen metagenome; EST-24 from *Bacillus paralicheniformis* T7; WP_326139525 from *B. halotolerans*; WP_268350347 from *B. haynesii*; WP_414557567 from *B. swezeyi*; WP_413544555 from *B. velezensis*; and WP_075622364 from *B. safensis*. Conserved motifs are highlighted. Residues strictly conserved among groups are shown in white font on red background. Four conserved blocks of I, II, III, and V are bracketed. Green triangles at the top of the alignment represent the location of the SGNH. The possible catalytic triads (serine (S), aspartic acid (D), histidine (H)) are shown at the bottom of the alignment with blue asterisks. Symbols above sequences represent the secondary structures; springs represent α-helices, and arrows represent β-sheets based on 6MKD_1 retrieved from PDB (pdb_00006nkd).

**Figure 6:**
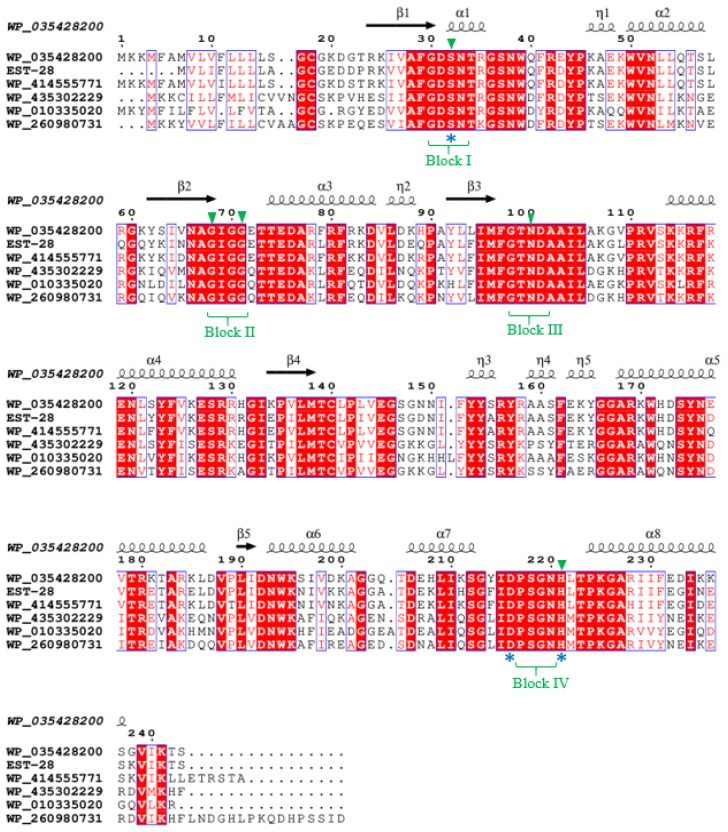
Amino acid sequence alignment analysis of EST-28. Sequences retrieved from National Center for Biotechnology Information (NCBI) server and aligned in Clustal W and rendered using ESPript output. WP_035428200 from multispecies of *Bacillus*; EST-28 from *B. paralicheniformis* T7; WP_414555771 from *B. swezeyi*; WP_435302229 from *B. maginnsis*; WP_010335020 from multispecies of *Bacillus*; and WP_260980731 from *B. pumilus*. Conserved motifs are highlighted. Residues strictly conserved among groups are shown in white font on red background. Four conserved blocks of I, II, III, and V are bracketed. Green triangles at the top of the alignment represent the location of the SGNH. The possible catalytic triads (serine (S), aspartic acid (D), histidine (H)) are shown at the bottom of the alignment with blue asterisks. Symbols above sequences represent the secondary structures; springs represent α-helices, and arrows represent β-sheets based on WP_035428200 retrieved from NCBI (A0A6I7FER0).

In this study, recombinant GDSL esterases were obtained from *B. paralicheniformis* T7 and their biochemical properties evaluated. Two GDSL esterases were identified in the *B. paralicheniformis* T7 genome: an esterase with a molecular weight of 26.8 kDa and a signal peptide (MVLIFLLLLAG) in the secretory proteome of the strain; and intracellular esterase with a molecular weight of 23.4 kDa. The open reading frames of EST-24 and EST-28 contain 212 and 237 amino acid residues, respectively. Multiple alignment of the EST-24 and EST-28 sequences with other bacillary GDSL esterases/lipases revealed that EST-24 and EST-28 possesses a conserved GDSX motif near the N-terminus of the protein (Figure 5 and Figure 6). The four invariant essential catalytic residues Ser, Gly, Asn, and His in blocks I, II, III, and V of EST-24 and EST-28, respectively, confirm their classification as SGNH hydrolase superfamily members. The catalytic triad of serine, aspartate, and histidine for EST-24 is located in blocks I and V at positions Ser11-Asp186-His189. In EST-28, this triad is located at positions Ser26-Asp200-His205.

Two recombinant analogues of these esterases were obtained via cloning: rEST-24, an analogue of the intracellular GDSL esterase; and rEST-28, an analogue of the extracellular GDSL esterase, but without the secretory peptide. Considering that the open reading frame in the pET-28/EST-24 and pET-28/EST-28 vectors contains 6× His-tag along with additional amino acid residues, the molecular mass of the recombinant rEST-24 and rEST-28 proteins changed to 26.1 kDa and 27.9 kDa, respectively.

Among bacillary esterases, exist monomeric proteins, such as enzymes [17,18,33,34,35,36,37,38,39,40,41,42,43,44,45,46], dimers [44,47], and oligomers [48] of subunits (Table 4). In terms of size, esterases are medium-sized proteins. Specifically, the molecular weight of monomeric bacillary esterases ranges from 24.5 kDa for and 25.0 kDa for *Bacillus licheniformis* [33] to 55.0 kDa for *Bacillus megaterium* WZ009 [34] and 60.3 kDa for *Bacillus subtilis* DR8806 [46]. The esterases EST-24 and EST-28 from *B. paralicheniformis* T7 also fall within this molecular weight range and are monomeric proteins.

Comparative analysis of the biochemical characteristics of esterases revealed that both esterases are neutral enzymes that display maximum activity at a pH of 7.0. Additionally, rEST-28 esterase exhibits activity under slightly acidic conditions. Both esterases exhibit maximum activity at 40 °C; however, unlike rEST-24, which is completely inactivated at 70 °C, rEST-28 remains active up to 80 °C. Similarly, most bacillary esterases are neutral or alkaline enzymes. However, the CarEW esterase from *Bacillus* sp. K91 is an acid esterase [35]. The temperature range at which *Bacillus* esterases exhibit maximum activity varies from 25 °C for esterases from *B. megaterium* WZ009 [34] to 65 °C for *B. subtilis* WB600 [49], *Bacillus acidocaldarius* [37,38], and *Bacillus* sp. 4 esterases [42]. Comparing the temperature indices of bacillary esterases with those of esterases from other genera revealed no pronounced differences; thus, an optimal temperature of 45 °C was established for the esterase from *Alteromonas* sp. 39-G1 [3], whereas the esterase from *Geobacillus* sp. JM6 [50] demonstrated maximum activity at 60 °C.

Bacillary GDSL esterases are characterised by their resistance to denaturation under pH fluctuations. Some retain over 80% of their maximum activity across a relatively wide pH range, rendering them ideal for use in changing chemical environments [17]. The evaluation of the pH stability of rEST-24 and rEST-28 revealed differences between the two enzymes. Following a 2 h incubation period under acidic (pH 4.0), neutral (pH 7.0), and alkaline (pH 10.0) conditions, rEST-24 retained no more than 25% of its esterase activity. However, rEST-28, exhibited greater stability, ranging from 35% under acidic pH to 120% under alkaline pH. The greater stability of rEST-28 under alkaline conditions may be attributed to the fact that it is an extracellular enzyme of *B. paralicheniformis* T7 that is capable of growing in a medium with a pH of 6.0–8.5. In contrast, the rEST-24 esterase functions inside the cell, where cytoplasmic pH fluctuations are less pronounced.

In addition, bacillary GDSL esterases are stable under fairly harsh temperature conditions. Thermostability studies show that many of these esterases retain optimal activity at temperatures between 50 and 60 °C and, in some cases, retain noteworthy catalytic function even after prolonged incubation at elevated temperatures [17]. This thermal stability is attributable to the stable structure of the α/β-hydrolase, which consists of a network of hydrogen bonds and hydrophobic interactions that strengthen the three-dimensional structure as a whole. The two GDSL esterases, rEST-24 and rEST-28, exhibited virtually identical thermal stability. Specifically, 1 h incubations at 40 °C and 50 °C reduced activity by no more than 40%, whereas after 2 h, residual activity was reduced to no more than 20%. Furthermore, incubation at 60 °C did not depend on the length of the incubation period; in both cases, the enzymes retained approximately 12% of their residual activity.

The esterases rEST-24 and rEST-28 are sensitive to divalent ions. These metal ions generally reduced the activity of the esterases in this study; however, rEST-24 exhibited tolerance to Mg^2+^, and the addition of 10 mM Mn^2+^ increased its activity. Copper and iron ions strongly inhibited rEST-24; however, rEST-28 activity remained at 33–34% despite being reduced. Furthermore, 10 mM DTT and β-mercaptoethanol completely inhibited rEST-28 activity, and rEST-28 exhibited resistance to non-ionic detergents, such as Tween-20, Tween-80, and Triton X-100. Given that rEST-24 is an intracellular enzyme and rEST-28 is an extracellular enzyme, the relative resistance of rEST-28 to detergents and inhibitors (except DTT and β-mercaptoethanol) may be attributed to its extracellular localisation. The same Mg^2+^ tolerance of rEST-24 was previously noted for esterases from *Bacillus aerophilus*, *B. pumilus* W3, and *B. velezensis* SYBC H47 [43,45,47]. The inhibitory effect of Cu^2+^ and Fe^2+^ ions was also established for the esterase of *B. subtilis* DR8806 [46]. As with rEST-24, resistance to 1% Triton X-100 was noted for the same esterase [46].

The substrates for bacillary esterases are fatty acid esters with short hydrocarbon chains [7]. Depending on the number of carbon atoms in the chain, the substrates are acetates (C_2_) [18,32,33,36,46,47,48], butyrates (C4) [32,33,39,42,47], or hexanoates (C6) [47] (Table 4). Analysis of the substrate specificity of rEST-24 and rEST-28 (Table 3) showed that rEST-28 is more specific to short-chain substrates (C2–C6) and is a true esterase, whereas rEST-24 demonstrates specificity to long-chain substrates (C10–C14), which may indicate its lipase activity. Previously, two enzymes with different substrate specificities were discovered in *Geobacillus thermocatenulatus* KCTC 3921. The enzyme Est29, which showed the highest activity on *p*NP-octanoate (C8), was classified as an esterase, whereas Lip29, which was more active on *p*NP-myristate (C14), was identified as a lipase [51]. A comparative analysis of V_max_ and K_m_ shows that the maximum reaction rate for bacillary esterases varies considerably, ranging from 0.22 U/mg for LipJ from *Bacillus* sp. JR3 [39] to 1449 U/mg for esterase from *B. subtilis* DSM402 [36] (Table 5). In this sstudy, esterases rEST-24 and rEST-28 exhibited average V_max_ values. *Bacillus* esterases are characterised by high substrate affinity, as evidenced by low K_m_ values (Table 5), thus suggesting that *B. paralicheniformis* T7 esterases require more substrate to achieve the maximum reaction rate.

Fermenting *B. paralicheniformis* T7 on four different media showed that salt medium supplemented with Tween-20 and YEPT with tributyrin as an esterase expression stimulator (and the sole carbon source) was ineffective as a nutrient medium for fermenting the producer strain. Nutrient broth or feather medium supplemented with 0.2% yeast extract were more effective. After four days of fermentation, esterase activity on these media was 163 ± 6 and 150 ± 3 U/mL, respectively. These results suggest that a medium enriched with peptides and amino acids, such as those found in meat peptone or formed through the hydrolysis of feather keratin, would be ideal for esterase synthesis. [21]. The level of esterase expression resulting from the fermentation of *B. paralicheniformis* T7 is considered adequate. Notably, this activity is 15–16 times higher than that observed in the *B. subtilis* E9 strain (10 U/mL) [40]. Moreover, lyophilization produced an enzyme preparation with an esterase activity of 17,618 ± 610 U/g. These results confirm the potential of using a feather medium to produce GDSL esterases via fermentation with the *B. paralicheniformis* strain.

## 5. Conclusions

Overall, *B. paralicheniformis* T7 can be used to produce esterase enzymes, and these new GDSL esterases from *B. paralicheniformis* T7 show promising potential in technologies for the hydrolysis and transesterification of fatty acid esters. Given the specificity of EST-28 for short-chain fatty acids, the enzyme shows great promise in technologies for the biocatalytic synthesis of acetic and butyric acid esters, which are used as flavorings in the perfume and food industries. In contrast, EST-24 appears to show promising potential for use in technologies for the hydrolysis and transesterification of fats and oils, including in the production of biodiesel.

## Figures and Tables

**Table 1:** Oligonucleotides for cloning.

Name	Sequence in 5′–3′ Direction
EST-24fw	GGGAATTCCATATGTGCGGAGAGGACGATCCC
EST-24rv	CGCGGATCCTCAAGAAGTTTTTATAACTTTACTTTCAT
EST-28fw	GGGAATTCCATATGTGCGGAGAGGACGATCCC
EST-28rv	CGCGGATCCTCAAGAAGTTTTTATAACTTTACTTTCAT
T7fw	TAATACGACTCACTATAGGG
T7rv	GCTAGTTATTGCTCAGCGG

**Table 2:** Effect of metal ions and detergents on esterase activity of rEST-24 and rEST-28.

Metal Ion	Concentration	Residual Activity, %	
		rEST-24	rEST-28
Control	10 mM	100 ± 5	100 ± 1
Ca^2+^	10 mM	52 ± 6	14 ± 1
Mg^2+^	10 mM	102 ± 3	52.3 ± 0.5
Mn^2+^	10 mM	126 ± 5	37.7 ± 0.6
Cu^2+^	10 mM	0	32.7 ± 0.5
Zn^2+^	10 mM	23 ± 4	33 ± 3
Fe^2+^	10 mM	0	34 ± 3
EDTA	10 mM	78 ± 2	54.5 ± 0.7
DTT	10 mM	12 ± 1	0
β-Mercaptoethanol	10 mM	37 ± 1	0
Tween-20	0.1%	54 ± 1	80.0 ± 0.6
Tween-80	0.1%	64 ± 2	74 ± 0.6
Triton X-100	0.1%	90 ± 5	46 ± 2
SDS	0.1%	30 ± 2	89 ± 3

All measurements were performed three times independently, and the average of the three replicates was reported as the defined result ± standard deviation (±SD). EDTA, ethylenediaminetetraacetic acid; DTT, dithiothreitol; SDS, sodium dodecyl sulfate.

**Table 3:** Substrate specificity of rEST-24 and rEST-28.

Substrate	Abbreviation	Specific Activity, U/mg	
		rEST-24	rEST-28
4-Nitrophenyl acetate (C2)	*p*NPA	16 ± 1	250.3 ± 0.1
4-Nitrophenyl butyrate (C4)	*p*NPB	39 ± 2	47.8 ± 0.2
4-Nitrophenyl hexanoate (C6)	*p*NPH	39 ± 2	93 ± 1
4-Nitrophenyl octanoate (C8)	*p*NPO	44 ± 2	24.4 ± 0.2
4-Nitrophenyl decanoate (C10)	*p*NPD	60 ± 2	31.1 ± 0.2
4-Nitrophenyl dodecanoate (C12)	*p*NPL	52 ± 1	42.3 ± 0.2
4-Nitrophenyl myristate (C14)	*p*NPM	51 ± 1	15.6 ± 0.2
4-Nitrophenyl palmitate (C16)	*p*NPP	45 ± 4	11.1 ± 0.1
4-Nitrophenyl stearate (C18)	*p*NPS	31 ± 16	32.2 ± 0.4

**Table 4:** Characteristics of bacillary esterases.

Enzyme	Source	Weight, kDa	Specific Activity, U/mg	Optimal pH	Optimal Temperature, °C	Reference
rEST-28	*Bacillus paralicheniformis* T7	27.9	193	7.0	40	this study
rEST-24	*Bacillus paralicheniformis* T7	26.1	250	6.0–7.0	40	this study
Est700	*Bacillus licheniformis*	25	no data	8.0	30	[33]
Est8	*Bacillus* sp. K91	24.5	no data	9.0	50	[17]
EST	*Bacillus megaterium* WZ009	55	135.3	11.5	25	[34]
BaCEs04	*Bacillus velezensis* SYBC H47	31.9 (monomer), 63.8 (dimer)	0.55	7.5	60	[47]
Esterase	*Bacillus pumilus*	17 (monomer), 170 (oligomer)	67.7	8.0	37	[48]
CarEW	*Bacillus* sp. K91	54	no data	4.5	45	[35]
AE6L	*Bacillus* sp. HR21-6	26	39.64	7.3	37	[18]
BS2	*Bacillus subtilis* DSM402	54	218	8.0–9.0	50	[36]
EST2	*Bacillus acidocaldarius*	34	1278	7.1	65	[37,38]
LipJ	*Bacillus* sp. JR3	46	0.05	7.0	30	[39]
EstE9	*Bacillus subtilis* E9	45	384	7.0	40	[40]
Esterase	*Bacillus cereus* WZZ001	55	no data	7.0	40	[41]
Esterase	*Bacillus* sp. 4	no data	833.33	6.0	65	[42]
Esterase	*Bacillus aerophilus*	28	238	8.0	37	[43]
Esterase I	*Bacillus subtilis* NRRL 365	36	80	8.0	30	[44]
Esterase II	*Bacillus subtilis* NRRL 365	57 and 48 (monomer), 105 (dimer)	520	8.0	30	[44]
BpFae12	*Bacillus pumilus* W3	35	12.8	8.0	50	[45]
Esterase	*Bacillus subtilis* DR8806	60.3	369	8.0	50	[46]

**Table 5:** Kinetic parameters of bacillary esterases.

Esterase	V_max_, U/mg	K_m_, mM	Substrate	Reference
EST-24 from *Bacillus paralicheniformis* T7	23.2	11.5	*p*NP decanoate (C10)	this study
EST-28 from *Bacillus paralicheniformis* T7	52.4	26.2	*p*NP acetate (C2)	this study
Esterase from *Bacillus subtilis* DR8806	151	4.2	*p*NP acetate (C2)	[46]
Esterase from *Bacillus pumilus*	49.02	3.94	*p*NP acetate (C2)	[48]
Est700 from *Bacillus licheniformis*	63.04	2.11	*p*NP acetate (C2)	[33]
Est19 from *Bacillus* sp. K91	277.78	0.42	*p*NP acetate (C2)	[32]
BaCEs04 from *Bacillus velezensis* SYBC H47	2.63	0.68	*p*NP acetate (C2)	[47]
AE6L from *Bacillus* sp. HR21-6	13.6	1.7	*p*NP acetate (C2)	[18]
Esterase from *B. subtilis* DSM402 (BS2)	1449	119	*p*NP acetate (C2)	[36]
Esterase from *Bacillus* sp. 4	833.33	0.063	*p*NP butyrate (C4)	[42]
Est700 from *Bacillus licheniformis*	22.76	1.38	*p*NP butyrate (C4)	[33]
Est19 from *Bacillus* sp. K91	17.86	0.48	*p*NP butyrate (C4)	[32]
BaCEs04 from *Bacillus velezensis* SYBC H47	6.87	0.73	*p*NP butyrate (C4)	[47]
LipJ from *Bacillus* sp. JR3	0.22	1.7	*p*NP butyrate (C4)	[39]
BaCEs04 from *Bacillus velezensis* SYBC H47	1.88	0.55	*p*NP hexanoate (C6)	[47]

## Data Availability

All datasets used and/or analyzed during the current study are available from the corresponding author on reasonable request.

## Abbreviations

The following abbreviations are used in this manuscript:

YEPT	yeast extract peptone medium
LB	Luria–Bertani
Ni-NTA	nickel-nitrilotriacetic acid
SDS	sodium dodecyl sulfate
PAGE	polyacrylamide gel electrophoresis
*p*NP	4-nitrophenol
DTT	dithiothreitol
EDTA	ethylenediaminetetraacetic acid
K_m_	Michaelis constant
V_max_	maximum velocity
SD	standard deviation
